# The Degree of Hydronephrosis as an Indicator of the Necessity for Ureteric Dilatation during Ureteroscopic Lithotripsy

**DOI:** 10.3390/jcm12144591

**Published:** 2023-07-10

**Authors:** Hyun-Soo Lee, Seon-Beom Jo, Wonku Hwang, Jong-Wook Kim, Mi-Mi Oh, Hong-Seok Park, Du-Geon Moon, Sun-Tae Ahn

**Affiliations:** Department of Urology, Korea University Guro Hospital, No. 148, Gurodong-ro, Guro-gu, Seoul 08308, Republic of Korea

**Keywords:** rigid ureteroscopic lithotripsy, difficult ureter, hydronephrosis

## Abstract

During rigid ureteroscopic lithotripsy, it is often encountered that the ureter is difficult to access. Attempts to advance the ureteroscope make the surgery more difficult. This study evaluated the preoperative predictive factors associated with difficult ureteral access (difficult ureter (DU)) during URS and assessed if clinical outcomes differed according to the degree of DU. This study identified 217 patients who underwent rigid ureteroscopic (URS) lithotripsy for the management of ureter stones between June 2017 and July 2021 in a tertiary hospital in Korea. In this group, preoperative factors were identified using univariate and multiple logistic regression analyses that could predict the degree of DU. Additionally, we also evaluated differences in treatment outcomes depending on the degree of DU. In 50 URS cases (22.0%), ureteral access using a ureteroscope was difficult. In the univariate and multivariate analyses, the degree of hydronephrosis was associated with the degree of DU. Treatment outcomes, extended operation times, low stone-free rate, postoperative pain, and secondary treatment were also significantly associated with the degree of DU. Clinicians can counsel patients with a lesser degree of hydronephrosis and approach their management accordingly.

## 1. Introduction

The global incidence of urolithiasis is changing, and its prevalence is increasing across the world [1]. In parallel, this has also been increasing in Korea since 1994 [2]. Because many urinary stones do not pass spontaneously, therapeutic interventions are often required [3].

There are various treatment methods for urolithiasis, such as medical expulsion, extracorporeal shock-wave lithotripsy (ESWL), and ureteroscopic lithotripsy (URS) [4]. In URS, as the ureteroscope is designed to account for rigidity, semi-rigidity, and flexibility, its diameter decreases [5], and stone-free rates with this technique are superior to those obtained with ESWL in urolithiasis [6]; hence, retrograde ureteroscopy has become the treatment of choice for urinary tract stones of less than 2 cm [7]. Despite advancements in ureteroscopy, it is still difficult to use ureteroscopes to access the ureter or the sheath during retrograde ureteroscopy [8]. The reports are variable, with difficulty in accessing ureters using retrograde ureteroscopes ranging from 8 to 40% [9,10]. Currently, there are no standard procedures for accessing such difficult ureters (DUs). Thus, additional procedures for ureteral dilatation are required in consultation with the surgeon [9]. DU may increase the risk of surgical failure, ureteral injury, and stricture [11,12].

Previous studies have attempted to predict DU; however, the limitation was that the risk associated with DU was different in each study [13,14,15], because the size and type of ureteroscope used and the definitions of difficult ureter were different for each study and even within individual studies. Additionally, previous studies did not analyze any statistical differences in treatment outcomes and complications, as per the presence of DU. Therefore, it is necessary to clearly define DU and reduce this bias by unifying the ureteroscope used during the study to predict the risk factors associated with DU based on preoperative characteristics. This study aimed to identify the risk factors associated with DU, which could reduce such existing biases and determine whether there are significant differences in treatment outcomes and complications in DU.

## 2. Materials and Methods

The study was conducted in accordance with the Declaration of Helsinki principles and was approved by the Institutional Review Board (or Ethics Committee) of Korea University Guro Hospital (2022GR0517; 23 December 2022).

### 2.1. Patient Data

This retrospective study was conducted between June 2017 and July 2021. Patients aged > 18 years, with ureteral stones, who underwent rigid ureteroscopic lithotripsy as the first treatment were included. In individuals with bilateral ureteroscopic lithotripsy, we considered each ureter procedure a separate event. Patients with known ureteral strictures, urinary tract abnormalities (such as ureterocele, ureter duplication, and horseshoe kidney), or ureterovesical junction (UVJ) stones that were closely located at the ureteral orifice with negligible access to the ureter were excluded. We also excluded patients presenting with acute stone-related symptoms, such as urinary tract infection (UTI) and acute renal injury. Their detailed medical history was obtained, including age, body mass index (BMI (kg/m^2^)), underlying comorbidities (diabetes mellitus (DM), prior history of ureteroscopic lithotripsy, pelvic surgery, or radiation), UTI episodes in the previous 12 months, and a history of medications in the form of steroids, anticoagulants, or antiplatelet agents. Radiological data were also collected using preoperative computed tomography (CT), including the location, size, number, and degree of hydronephrosis (HN). When there were multiple stones, their size and location were determined based on the largest stone. The degree of HN was assessed based on CT findings, and it was classified as none, mild degree, or moderate-to-severe degree according to the radiology grading system [16], with a score of 2 being mild degree and 3 or higher being moderate-to-severe degree.

### 2.2. Surgical Procedure and Postoperative Management

All the surgical procedures were performed at a tertiary hospital in Korea. Under general anesthesia, an 8/9.8-Fr Wolf (Richard Wolf, Knittlingen, Germany) rigid ureteroscope, a ureter balloon dilator (Boston Scientific, Natick, MA, USA), and endoscopic scissors (Storz, Tuttlingen, Germany) were used for surgery. A stone laser and a basket were also used. The ureteroscope was inserted into the ureteral orifice using a guidewire. If access to the ureter was difficult, retrograde pyelography (RGP) was used to identify a narrow point. In case of the pinned point narrowing on RGP, endureterotomy was performed; otherwise, balloon dilatation was performed. The stone was crushed using a stone laser, and the remaining stone fragments were removed using a stone basket or forceps. Subsequently, a double-J catheter was inserted. At the end of the procedure, the operation time was recorded, and postoperative residual stones and ureter injuries were evaluated. All surgeries were performed using the abovementioned procedures and materials. Two–four weeks after the surgery, the patients were followed up on an outpatient basis, and postoperative pain, UTI, and gross hematuria were evaluated. In cases of residual stones, secondary treatment was planned if there was no spontaneous expulsion.

### 2.3. Outcome Assessment

Patients with narrow ureters who required ureteral dilatation were classified into the DU group, and those without narrow ureters were classified into the non-DU group. The primary outcome was the incidence of DU, defined as the inability to access stones above the UVJ level owing to narrow ureter that would, otherwise, require dilation to advance the ureteroscope. As a primary outcome, we also assessed the risk factors for DU. Baseline characteristics were compared between the DU and non-DU groups to evaluate the risk factors for DU. The secondary outcome was assessed based on whether there were significant differences in treatment outcomes and complications between the DU and non-DU groups.

### 2.4. Statistical Analysis

Continuous data were analyzed using Student’s *t*-test or Mann–Whitney U test based on the distribution of the data. All continuous data are presented as means ± standard deviations (SDs). Categorical variables were examined using Pearson’s chi-square test, where appropriate (expected frequency > 5); otherwise, Fisher’s exact test was used. Categorical data are expressed as numbers and percentages. The risk factors for DU were assessed using univariate logistic regression analysis. The risk factors in the univariate analyses (*p* < 0.20) were included in the multivariate analysis. In addition, the treatment results according to DU were analyzed using univariate logistic regression (odds ratio (OR); 95% confidence interval (CI)) All analyses were performed using IBM SPSS Statistics, ver. 26.0 (IBM Co., Armonk, NY, USA). Statistical significance was set at *p* < 0.05.

## 3. Results

### 3.1. Preoperative Characteristics between DU and Non-DU Groups

After reviewing the records, 258 patients were identified. Patients were >18 years old with ureteral stones and underwent rigid ureteroscopic lithotripsy as the initial treatment. Patients with known urinary tract abnormalities (n = 18), UVJ stones (n = 11), acute symptoms (n = 7), and pre-stenting (n = 5) were excluded. Among the remaining 217 eligible patients, 10 had bilateral stones. In total, 227 rigid ureteroscopic lithotripsy procedures were performed.

The overall incidence of DU was 22% (50/227) in our study. All patients with DU underwent intraoperative ureteral dilatation and postoperative stenting. The incidence of DU was significantly higher in the low-grade-HN group than in the other groups (*p* = 0.008). Demographic variables like age (*p* = 0.190) and sex (*p* = 0.413) did not differ. Except for HN, radiological factors, such as the number (*p* = 0.234), size (*p* = 0.386), and location (*p* = 0.244) of the stones, were not statistically significant. Clinical variables were also not statistically significant, and the *p*-values for each variable were as follows: BMI, *p* = 0.283; UTI, *p* = 0.812; previous ureteroscopic surgery, *p* = 0.170; previous pelvic surgery or radiation, *p* = 0.505; DM, *p* = 0.776; steroid use, *p* = 0.420; and antiplatelet or anticoagulant use, *p* = 0.847. The baseline demographic, radiological, and clinical characteristics of the DU and non-DU groups are summarized in Table 1.

### 3.2. Univariate and Multivariate Logistic Regression for Preoperative Characteristics between DU and Non-DU Groups

Univariate logistic regression analyses identified HN as a preoperative risk factor for DU (no HN, *p* = 0.006; mild HN, *p* = 0.012). Other variables did not show statistical significance; however, variables such as age (*p* = 0.183), previous ureteroscopic surgery (*p* = 0.177), and DM (*p* = 0.169) were significant and were analyzed again using multivariate analyses together with HN. In multivariate logistic regression analyses, the degree of HN remained a risk factor for DU (no HN, *p* = 0.013; OR, 2.899; 95% CI, 1.252–6.715; mild HN, *p* = 0.018; OR, 2.553; 95% CI: 1.176–5.541, using moderate-to-severe HN as reference value). The other variables were not statistically significant. The results of the logistic regression analysis are summarized in Table 2.

### 3.3. Treatment Outcomes and Complications in DU and Non-DU Groups

In terms of treatment outcomes, the operation time was significantly longer in DU than in the non-DU condition (*p* < 0.001). The stone-free rate was also significantly lower in the DU group than in the non-DU group (OR, 13.66; 95% CI, 5.27–35.36; *p* < 0.001). The necessity for secondary treatment with residual stones was also significantly higher in DU than in the non-DU condition (OR, 16.03; 95% CI, 5.90–43.49; *p* < 0.001). Complications were resolved with conservative treatment. A statistically significant complication was postoperative pain (OR, 3.822; 95% CI, 1.060–13.77; *p* = 0.029), which was higher in the DU group than in the non-DU group. Ureter injury, length of hospital stay, UTI, and gross hematuria were not statistically significant. The treatment outcomes are summarized in Table 3.

## 4. Discussion

In this study, the prevalence and risk factors of DU during rigid ureteroscopic lithotripsy were evaluated. This study also evaluated the treatment outcomes and complications in patients with DU, which were previously unaddressed. The prevalence of DU was estimated at 22%, and a lesser degree of HN was reported as a significant predictor of DU. Additionally, in terms of treatment outcomes and complications, patients with DU showed longer operation times, lower stone-free rates, more postoperative pain, and higher need for secondary treatment. These results may allow clinicians to predict which patients are at high risk of DU based on the severity of HN, allowing them to better anticipate intraoperative complications and prepare accordingly. In counseling, treatment results are poorer in patients with DU than in non-DU patients, which can reduce patient satisfaction. Clinicians can set realistic treatment outcomes in consultation with the patients by discussing the potential surgical difficulties and the need for additional treatment. Other studies have reported the rate of failure in accessing stones, with pre-stenting as a requirement, to vary between 8 and 15% [8]. In a large-scale study conducted by Castro et al. on 9681 patients undergoing ureteroscopic stone treatment, the rates of balloon dilatation varied from 21% to 40% [10]. The ratio of DU in previous studies was considered to be similar. We demonstrated that reduced HN is associated with DU. This can be explained by the fact that HN is a dilatation due to the increase in pressure caused by obstructive uropathy [17]. In patients with low-grade HN, it is thought that these ureters do not tend to dilate even with pressure, that is, low-grade HN may be related to a lesser tendency for ureter dilation during ureteroscopy, which makes it difficult to advance the ureteroscope and is thought to be associated with DU. The identification of a lesser degree of HN as a risk factor for difficult ureter (DU) is thought to be related to the pathologic change in the ureter itself. In previous studies dealing with ureter pathology in difficult ureter [18,19], the pathology of ureter stricture has been reported to be related to inflammation, fibroplasia, hyalinization [18], polyp or granulation tissue growth secondary to stone, or other benign or neoplastic cause stricture [19]. In this study, a lesser degree of HN was identified as a risk factor for difficult ureter (DU), which is considered to be related to pathologic changes in the ureter itself, not to stone-related factors or clinical factors. Although additional studies are needed, a low degree of HN can also be considered an indicator of ureter pathologic changes, as shown in the studies [18,19]. In addition, HN has been identified as a risk factor for DU, making surgery more difficult and resulting in worse outcomes. Preoperative imaging is necessary to confirm the degree of HN. As for the imaging modality, it is necessary to CT because it is more accurate than renal ultrasound [20,21], intravenous urography [22], or kidney–ureter–bladder (KUB) radiography [23] in evaluating HN or stones. Identifying HN preoperatively using imaging techniques could help plan surgical methods and use appropriate equipment.

Other studies have reported younger age, absence of stone history, renal stones [13], large stones [14], small stones, smoking, and absence of DM [15] as risk factors for DU. Unlike previous studies, the present study reported that less severe HN was a risk factor for DU. These studies have revealed different risk factors, and this can be partially explained by the differences in surgical instrument and method used. In this study, only a rigid ureteroscope was used; however, in previous studies, a flexible scope and semirigid ureteroscope were used together. In this study, as in the study dealing with the pathophysiology of the ureter stricture itself [18,19], difficult ureter (DU) was thought to be related to the pathology of the ureter itself rather than a clinical, epidemiologic, or stone-related factor. This is why the results are different from previous studies dealing with difficult ureter (DU) [13,14,15]. Thus, it would be worthwhile to study the integration of other clinical indicators.

We also analyzed treatment results and outcomes, which were not analyzed in previous studies. The DU group had a significantly longer operation time and lower stone-free rate. The longer operation time is thought to be due to the need for additional procedures, such as RGP and ureter dilation in the DU. These additional processes increase the difficulty of the surgery, which may reduce the stone-free rate. As postoperative outcomes, pain and need for additional treatment were also higher in the DU group. This is likely due to the effect of remnant stones, because the stone-free rate was significantly lower in the DU group. However, the risk of ureteral injury was not significantly different between the DU and non-DU groups, which could guarantee the safety of ureter dilatation [24].

The limitations of this study include its retrospective design and small sample size. That is, selection bias may have occurred because of the retrospective design. To minimize this, definite medical records were used, and subjective factors that could occur during group selection were eliminated by setting clear inclusion and exclusion criteria. Additionally, there is a possibility of bias due to the surgical results of a single center and surgeon, and if other surgical techniques and equipment were used, we could not rule out the possibility of variations in the results. Some other limitations of this study, which are also similar to those of previous studies, include dealing with DU, which may have resulted in different risk factors for DU. This can be complemented by future prospective studies.

## 5. Conclusions

In this study, the rate of DU was about 22%, and low-grade HN was identified as a risk factor for DU. Additionally, it was confirmed that the longer operation time in the DU group placed a burden on the surgeon, and in terms of treatment results, the stone-free rate was low, and the patient’s satisfaction was reduced because additional treatment was required. Therefore, it is necessary to predict DU in advance according to the degree of HN, and realistic treatment results need to be discussed between clinicians and patients. In addition, to overcome the limitations of this study, it is necessary to reconfirm the risk factors for DU by reducing the bias of the outcome with a future multicenter, large-scale study in a prospective manner and comparing it with the risk factors reported in previous studies.

## Figures and Tables

**Table 1:** Comparison of clinical features of patients who needed/did not need ureter dilatation to access the ureter during rigid ureteroscopic lithotripsy.

		All (n = 227)	DU (n = 50)	Non-DU (n = 177)	*p*-Value
Demographic variables					
Age		59.97	62.06 (11.18)	59.39 (12.8)	0.190
Sex	Male	134	27 (54.0)	107 (60.5)	0.413
	Female	93	23 (46.0)	70 (39.5)	
Radiologic variables					
Hydronephrosis	No	50	16 (32.0)	34 (19.2)	0.008
	Mild	70	20 (40.0)	50 (28.2)	
	Moderate to severe	107	14 (28.0)	93 (52.6)	
Number of stones	Single	203	47 (94.0)	156 (88.1)	0.234
	Multiple	24	3 (6.0)	21 (11.9)	
Stone size		0.796	0.831 (0.312)	0.786 (0.337)	0.386
Stone location	UPJ	57	27 (28.0)	30 (16.9)	0.244
	Proximal	123	25 (50.0)	98 (55.4)	
	Mid	45	7 (14.0)	38 (21.5)	
	Distal	15	4 (8.0)	11 (6.2)	
Clinical variables					
BMI (kg/m^2^)		25.60	24.96 (2.96)	25.78 (5.10)	0.283
Recent UTI within 1 year	Yes	48	11 (22.0)	37 (20.5)	0.812
	No	179	39 (78.0)	140 (79.5)	
Previous ureteroscopic surgery	Yes	17	6 (12.0)	11 (6.2)	0.170
	No	210	44 (88.0)	166 (93.8)	
Pelvic operation or radiation	Yes	58	18 (36.0)	40 (22.6)	0.505
	No	169	32 (64.0)	137 (77.4)	
DM	Yes	58	12 (24.0)	46 (26.0)	0.776
	No	169	38 (76.0)	131 (74.0)	
Steroid use	Yes	9	1 (2.0)	8 (4.5)	0.420
	No	218	49 (98.0)	169 (95.5)	
Antiplatelet or anticoagulant use	Yes	43	9 (18.0)	34 (19.2)	0.847
	No	184	41 (82.0)	143 (80.8)	

BMI = body mass index; UTI = urinary tract infection; DM = diabetes mellitus; DU = difficult ureter.

**Table 2:** Univariate and multivariate logistic regression for preoperative characteristics between DU and non-DU groups.

Variables		Univariate			Multivariate		
		OR	95% CI	*p*-Value	OR	95% CI	*p*-Value
Demographic variables							
Age		1.018	0.992–1.044	0.183	1.019	0.992–1.047	0.160
Sex		1.302	0.692–2.451	0.413			
Radiologic variables							
Hydronephrosis	(moderate to severe as reference value)						
	Mild	2.657	1.237–5.708	0.012	2.553	1.176–5.541	0.018
	No	3.126	1.380–7.082	0.006	2.899	1.252–6.715	0.013
Number of stones		0.474	0.135–1.660	0.243			
Stone size		0.633	0.228–1.760	0.381			
Stone location		0.784	0.521–1.180	0.278			
Clinical variables							
BMI (kg/m^2^)		0.957	0.886–1.035	0.273			
Recent UTI within 1 year		1.060	0.495–2.268	0.881			
Previous URS		2.058	0.721–5.873	0.177	1.522	0.503–4.601	0.457
Pelvic operation or radiation		0.780	0.369–1.648	0.515			
DM		0.573	0.259–1.267	0.169	0.600	0.264–1.362	0.222
Steroid use		1.819	0.438–7.549	0.410			
Antiplatelet use		0.923	0.410–2.081	0.847			

BMI = body mass index; UTI = urinary tract infection; DM = diabetes mellitus; URS = ureteroscopic removal of stone; DU = difficult ureter; OR = odds ratio; CI = confidence interval.

**Table 3:** Results of treatment outcomes and complications in DU and non-DU groups.

	All (n = 227)	DU (n = 50)	Non-DU (n = 177)	*p*-Value
Operation time	36.96 (22.58)	56.94 (30.26)	31.31 (15.95)	<0.001
Stone-free rate	202/227 (88.9%)	32/50 (64%)	170/177 (96.0%)	<0.001
Ureter injury	1.7%	2 (4%)	2 (1.1%)	0.173
Hospital stay	1.28 (0.88)	1.32 (0.97)	1.27 (0.85)	0.455
UTI	6 (2.6%)	1 (2%)	5 (2.8%)	0.748
Gross hematuria	11 (4.8%)	2 (4%)	9 (5.0%)	0.752
Secondary treatment	24 (10.5%)	18 (36%)	6 (3.4%)	<0.001
Postoperative pain	10 (4.4%)	5 (10%)	5 (2.8%)	<0.029

UTI = urinary tract infection; DU = difficult ureter.

## Data Availability

Not applicable.
